# Advancing Design Strategy of PROTACs for Cancer Therapy

**DOI:** 10.1002/mco2.70258

**Published:** 2025-06-25

**Authors:** Hang Luo, Yuan Tian, Razack Abdullah, Baoting Zhang, Yuan Ma, Ge Zhang

**Affiliations:** ^1^ School of Chinese Medicine Faculty of Medicine The Chinese University of Hong Kong Hong Kong SAR China; ^2^ Law Sau Fai Institute for Advancing Translational Medicine in Bone &Joint Diseases, School of Chinese Medicine Hong Kong Baptist University Hong Kong SAR China; ^3^ Increasepharm & Hong Kong Baptist University Joint Centre for Nucleic Acid Drug Discovery Hong Kong, Science Park, New Territories Hong Kong SAR China

**Keywords:** artificial intelligence, cancer therapy, E3 ligand, linker design, POI ligand, proteolysis targeting chimeras (PROTACs)

## Abstract

Proteolysis targeting chimeras (PROTACs) have emerged as a groundbreaking class of anticancer therapeutics. These bifunctional molecules harness the endogenous ubiquitin–proteasome system to facilitate the degradation of targeted proteins of interest (POIs). Notably, the clinical translation of PROTACs has gained substantial momentum, with many PROTAC candidates targeting various cancers currently undergoing clinical trials (Phase I–III). However, the rational design of high‐efficacy PROTAC compounds remains a significant challenge. In this review, we presented a comprehensive overview of POI ligands, E3 ligands, and their interconnected linkers in PROTAC design, including their generation, structural optimization, and contribution to degradation efficiency and selectivity. Particularly, we analyzed the distinct preferences of various types of POI ligands (small molecule, nucleic acid, and peptide) toward specific targets. Furthermore, we emphasized the significant role of artificial intelligence technology in PROTAC design, including POI/E3 ligands discovery and linkers generation or optimization. We also summarized the applications and challenges of PROTACs in cancer therapy. Finally, we discussed the future development of PROTAC by combining multidisciplinary technologies and novel modalities for cancer therapy. Overall, this review aims to provide valuable insights for advancing PROTAC design strategies for cancer therapy.

## Graphical abstract

A comprehensive update on the PROTAC design, and highlighting the role of artificial intelligence in the PROTAC design for cancer therapy.

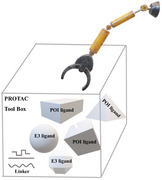

## 1 Introduction

Proteolysis targeting chimeras (PROTACs) are heterobifunctional molecules that consist of two different ligands connected by a linker [1]. These ligands in PROTACs can simultaneously bind to the proteins of interest (POIs) and the E3 ligase, facilitating the formation of a POI/PROTAC/E3 ligase ternary complex. Then, PROTACs hijack the ubiquitin–proteasome system (UPS) to degrade POI [2, 3]. Notably, PROTACs have demonstrated remarkable success in targeting various cancer‐related proteins such as androgen receptor (AR) [4, 5], estrogen receptor (ER) [6], bromodomain and extra‐terminal (BET) [7, 8, 9], Bruton's tyrosine kinase (BTK) [10, 11, 12, 13], breakpoint cluster region‐Abelson tyrosine kinase (BCR‐ABL) [14], signal transducer and activator of transcription 3 (STAT3) [15], anaplastic lymphoma kinase (ALK) [16], Kirsten rat sarcoma viral oncogene homologue (KRAS) [17, 18], and cyclin‐dependent kinase (CDK) 2/4/6/9 [19, 20, 21], highlighting the versatility and potential of PROTACs in cancer therapy. Several online PROTAC databases have also been published [22, 23, 24]. More importantly, the effectiveness of PROTAC technologies in cancer therapy is evident from the advancement of various PROTAC compounds into clinical trials (phases I‐III) [25, 26].

However, despite the progress made in PROTAC design, the current strategies largely rely on empirical approaches and extensive experiments [27, 28, 29]. Exploring the vast chemical space to identify ideal PROTAC compounds was challenging and time‐consuming. Recently, some artificial intelligence (AI)‐based PROTAC design strategies were developed, which could be used for accelerating the design process of PROTACs [30, 31, 32]. It paved the novel way for PROTAC design in cancer therapy [33]. Although there were many reviews related to PROTACs in cancer therapy, which mainly focused on therapeutical efficiency and application [34, 35, 36, 37]. It lacked the discussion and perspectives of advancing PROTAC design strategy.

In this review, we provided a comprehensive overview of POI ligands, E3 ligands, and their interconnected linkers in PROTAC design. The distinct preferences exhibited by different types of POI ligands toward specific targets were discussed. Importantly, the potential of AI technology in advancing PROTAC design strategies was highlighted. Subsequently, the applications and challenges of PROTACs in cancer therapy were summarized. Finally, the future directions of PROTACs in design and development were discussed. By improving our understanding of these design principles, we can pave the way for the development of more promising PROTACs for cancer therapy.

## 2 Mechanisms of Action and Components of PROTACs

**FIGURE 1:**
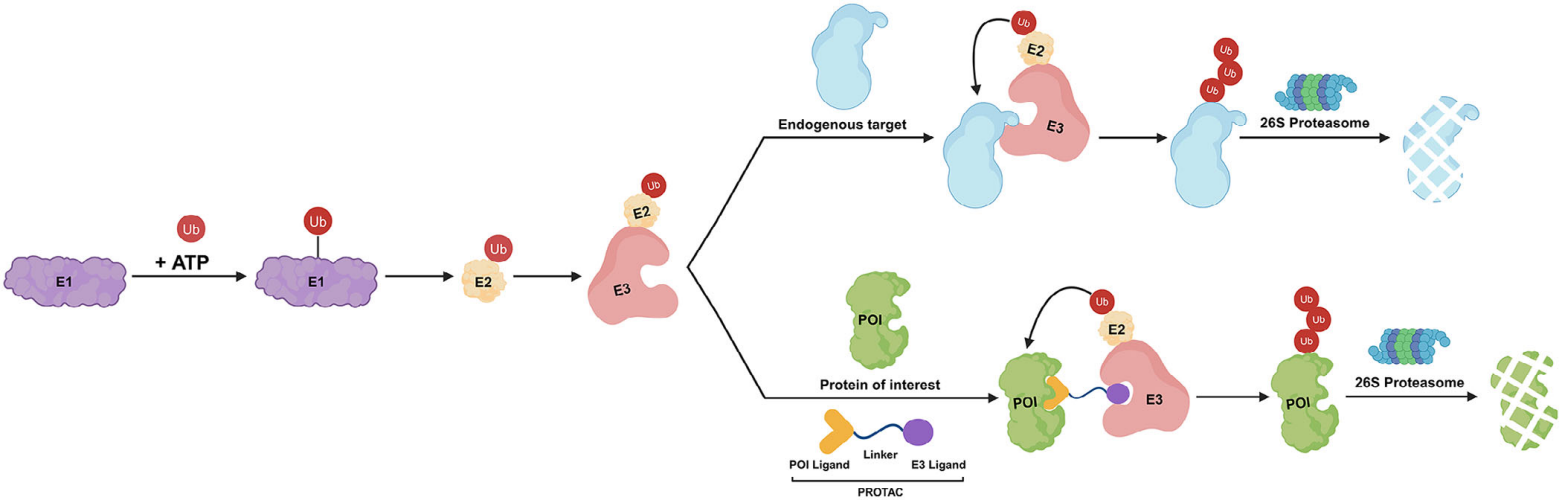
Mechanism of action of UPS system‐mediated endogenous protein degradation and PROTACs‐mediated targeted protein degradation. First, the E1 could activate ubiquitin in an ATP‐dependent manner. Subsequently, the ubiquitin could be transferred on E2, and then the E3–E2–ubiquitin complex could be formed. For the endogenous protein ubiquitination, the polyubiquitin could be labeled on the endogenous protein by E3 ligase. For the POI targeted ubiquitination, the polyubiquitin could be formed on the POI by E3 ligase catalysis after forming a POI/PROTAC/E3 ternary complex. Finally, the ubiquitinated endogenous protein or POI could be transported to proteasome for degradation. ATP: adenosine triphosphate. The Figure 1 was created in BioRender.com.

The UPS system is one of the important pathways for degrading intracellular proteins to maintain cell homeostasis [3]. The UPS system includes ubiquitin (Ub), three enzymes (E1 Ub‐activating enzyme, E2 Ub‐conjugating enzyme, and E3 ligase), proteasome, and specific substrates. E1 could activate Ub and then the Ub was transferred to E2. Subsequently, the Ub on E3 was further transferred to substrate proteins via E3 ligase. Based on the catalytical function of E3 ligase, the polyubiquitin chain would be formed on substrate proteins, and then the polyubiquitinated protein could be recognized by 26S proteasome and transported to 20S proteasome for further degradation (Figure 1) [38].

PROTAC consists of a POI ligand, a linker, and an E3 ligand. In the PROTAC‐mediated POI degradation process, the PROTAC could simultaneously bind POI and specific E3 ligase to form a ternary complex, and this spatial proximity allowed that the polyubiquitin was transferred on the POI for further degradation via proteasome (Figure 1). Particularly, the PROTAC could recycle after finishing one POI degradation cycle [39]. The effective POI degradation mainly depended on the stable ternary complex formation induced by PROTACs. In the ternary complex, POI or POI ligand could interact with E3 ligase, and E3 ligand could interact with POI. These intramolecular interactions were necessary for the formation and stability of the ternary complex [40]. So far, several crystallized structures of ternary complex induced by PROTACs have been reported [41, 42, 43, 44, 45]. Based on the crystallized structures, researchers could achieve precise PROTAC molecule modification or optimization [46]. The stability of the ternary complex induced by PROTAC could be quantified according to the cooperativity factor (*α*) [47]. The cooperativity factor (*α*) was defined as the ration of binary (POI/PROTAC or E3 ligase/PROTAC) and ternary (POI/PROTAC/E3 ligase) dissociation constants. When *α* > 1, it means that the ternary complex became more stable than the binary complex [48]. Currently, there are various approaches for measuring the cooperativity including AlphaScreen/AlphaLISA [2, 49], biolayer interferometry [50], surface plasmon resonance [47], and time‐resolved fluorescence resonance energy transfer [10]. Notably, some studies reported that the lysine accessibility on POIs played an important role in the PROTAC‐induced protein degradation [51]. For example, Ciulli's group [52] used cryo‐electron microscopy (cryo‐EM) structure data and in vitro ubiquitination assay reveled that the lysine 456 on Brd4^BD2^ could be an optimal position for ubiquitination reaction, which was critical for MZ1‐induced protein degradation. Moreover, they demonstrated that the importance for degradation of targets orientation and proximity to the RING activated E2‐Ub catalytic module of the Cullin‐RING ligase (CRL). The preferred distance and geometry would allow the lysine residues reacted conveniently with the Ub–E2 thioester. In contrast, the lysines on the disfavored position were less likely to be ubiquitinated. Overall, the comprehensive understanding of the mechanism of action for PROTACs was necessary for future development, which could guide us to refine rational PROTAC drug design.

## 3 Advances in Design Strategies of PROTACs

In the past decade, extensive efforts had been made for the development of PROTACs. The various PROTACs such as small molecule‐based PROTACs, nucleic acid‐based PROTACs, peptide‐based PROTACs were developed for cancer‐related targets degradation. In this section, we will overview the POI ligands, E3 ligands, and linker design in these PROTACs.

### 3.1 POI Ligand in PROTAC Design

#### 3.1.1 Small Molecule as POI Ligand in PROTAC Design

**FIGURE 2:**
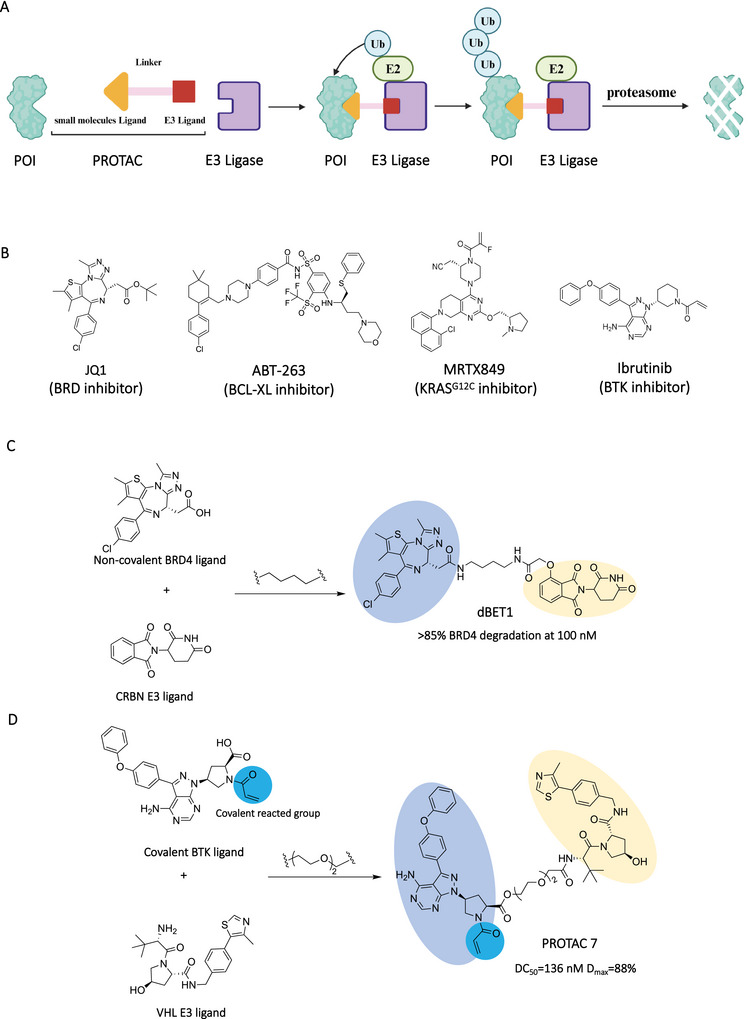
Small molecules as POI ligands in PROTACs. (A) The action mode of small molecules‐based PROTACs. (B) The chemical structures of representative small molecule POI ligands including JQ1, ABT‐263, MRTX849, and ibrutinib. (C) Design of noncovalent small molecule‐based PROTAC (dBET1) by connecting the noncovalent BRD4 ligand and a CRBN E3 ligand. (D) Design of covalent small molecule‐based PROTAC (PROTAC 7) by connecting the covalent BTK ligand and a VHL E3 ligand. The blue part represents the covalent reaction group, and the light blue part and light orange part represent POI ligand and E3 ligand, respectively. The Figure 2A was created in BioRender.com.

In the existed PROTACs, the majority of PROTACs used small molecules as the POI ligand, which was conveniently converted to PROTACs for further POI degradation (Figure 2A). These small molecule ligands mainly were established inhibitors such as JQ1 (bromodomain (BRD) inhibitor) [53], ABT‐263 (B‐cell lymphoma extra large (BCL‐XL) inhibitor) [54], and MRTX849 (KRAS^G12C^ inhibitor) [18], which had confirmed the structure‐activity relationships (SARs). Some of them were FDA‐approved drugs such as ibrutinib (BTK inhibitor) (Figure 2B) [55]. Through sample function group (─COOH, ─NH_2_, ─N_3_, and so on) transformation, these small molecules could be converted to the PROTAC compounds by connecting with the E3 ligands via linkers. For example, Winter et al. [56] removed the tertiary butyl in JQ1 by formic acid, and then it was connected to the CRBN (Cereblon) ligand with a linker to synthesize a high effective BRD4 PROTAC dBET1 (>85% degradation at 100 nM) (Figure 2C). Generally, the high binding affinity between POI and small molecule warhead was required for efficient POI degradation. Zhou et al. [15] designed the potent STAT3 PROTAC SD‐36 by converting the STAT3 inhibitor SI‐109 (*K*_i_ = 14 nM). Their results showed that SD‐36 kept binding affinity to STAT3 (KD = 44 nM). SD‐36 could effectively reduce the level of STAT3 in SU‐DHL‐1 cells (DC_50_ = 28 nM). Particularly, different small molecule ligands could be converted to PROTACs for the same POI degradation. The representative example was the design of BCR‐ABL PROTACs. BCR‐ABL protein has multiple binding pockets for small molecules, and researchers reported three BCR‐ABL PROTACs based on different inhibitors including dasatinib, asciminib, and ponatinib, which bond BCR‐ABL protein via different binding pockets. In 2016, the Crews group [57] first reported the dasatinib‐based PROTAC could induce effective BCR‐ABL degradation (>60% degradation at 1 µM). Subsequently, the Crews group [58] also reported the asciminib‐based PROTAC GMB‐805 for BCR‐ABL degradation (DC_50_ = 30 nM). Rao et al. [59] demonstrated that the ponatinib‐based PROTAC PI9P could significantly degrade BCR‐ABL (DC_50_ = 20 nM). It indicated the great potential of PROTAC to overcome target mutation and drug resistance.

The covalent inhibitors also were used for PROTAC design for those targets without high‐affinity noncovalent ligands [60, 61]. Xue et al. [62] developed a kind of covalent inhibitor‐based PROTAC that irreversibly covalently bound BTK with ibrutinib and recruited VHL (von Hippel–Lindau) E3 ligase for BTK degradation (Figure 2D). Their results showed that PROTAC significantly reduced BTK protein levels with a DC_50_ value of 136 nM and a *D*_max_ value of 88%. Buhimschi et al. [13] designed a reversibly covalent BTK PROTAC MTK802. Their results showed that MTK802 could induce the degradation of C481S mutant BTK and wild‐type BTK with DC_50_ values of 14.9 and 14.6 nM, respectively. Bond et al. [17] developed the PROTAC LC‐2 targeting KRAS, which was a frequently mutated protein in cancer. LC‐2 consisted of the covalent KRAS^G12C^ inhibitor MRTX849, a linker, and a VHL ligand. It could induce rapid degradation of KRAS^G12C^, resulting in inhibition of mitogen‐activated protein kinase (MAPK) signaling in both pure and heterozygous KRAS^G12C^ cells. They tested the degradation efficiency of LC‐2 in five different KRAS^G12C^ cells with DC_50_ values ranging from 0.25 to 0.76 µM.

Generally, cellular signaling was mediated by various multivalent interactions. This multivalency could allow stable biophysical recognition [63]. Similarly, multivalency was an effective strategy for drug design. In the past years, there have been many successful multivalent drug design cases such as bivalent small molecule inhibitors [53], trivalent N‐acetylgalactosamine [64], and bi‐specific antibodies [65]. For facilitating degradation efficacy and spectrum of PROTACs, multivalent PROTACs also were designed [66]. For instance, Zheng et al. [67] designed a dual PROTAC (DP‐C‐1), which consisted of two POI ligands, one E3 ligand and an amino acids‐based star‐type linker. Their results showed that DP‐C‐1 could simultaneously degrade epidermal growth factor receptor (EGFR) and poly‐ADP ribosepolymerase at micromolar concentration in cancer cells. Chen et al. [68] developed the dual‐ligand PROTACs that had two POI ligands, two E3 ligands, and a branched linker. Their results showed that dual‐ligand PROTAC (2J2V) exhibited a 10‐fold increase in degradation efficiency and a 100‐fold increase in cytotoxicity than single‐ligand PROTAC in cancer cells.

Overall, various small molecule‐based PROTACs successfully degraded various cancer‐related POIs for cancer therapy. In addition, based on the binding mode of small molecules and POIs, small molecule‐based PROTACs preferred to recruit the POIs with well‐defined pockets for further degradation.

#### 3.1.2 Nucleic Acid as POI Ligand in PROTAC Design

**FIGURE 3:**
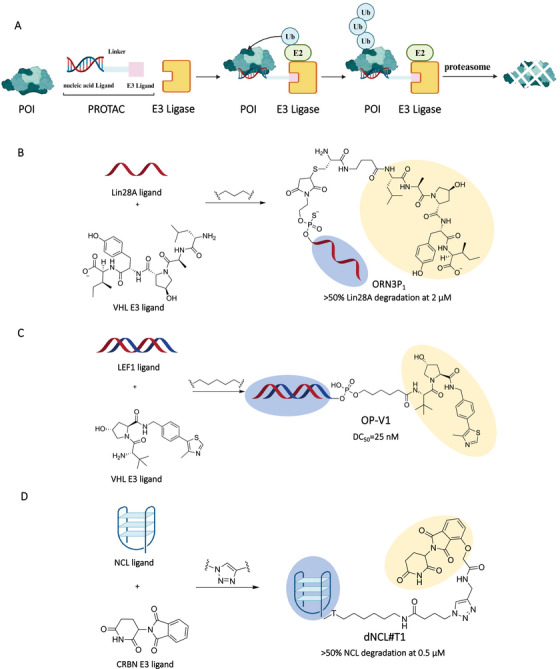
Nucleic acids as POI ligands in PROTACs. (A) The action mode of nucleic acid‐based PROTACs. (B) Design of single strand RNA‐based PROTAC (ORN3P_1_) by connecting the single strand RNA targeting Lin28A and a VHL E3 ligand. (C) Design of double strand DNA‐based PROTAC (OP‐V1) by connecting the double strand DNA targeting LEF1 and a VHL E3 ligand. (D) Design of aptamer‐based PROTAC (dNCL#T1) by connecting the aptamer targeting NCL and a CRBN ligand. The light blue part and light orange part represent POI ligand and E3 ligand, respectively. The Figure 3A was created in BioRender.com.

In cellular proteins, ∼80% of POIs had no specific binding pockets [69]. For example, there were lots of transcription factors (TFs) lacking specific binding pockets in cells, and mutations in these proteins can lead to various diseases, including cancers [70]. Therefore, it was challenging to target these proteins to inhibit cancer progress. These intractable proteins were regarded as undruggable targets [71]. Unlike small molecule‐based PROTACs, nucleic acid‐based PROTACs can bind POIs through specific sequences or their 2D/3D structural conformations for further degradation (Figure 3A) [72, 73, 74, 75, 76, 77]. In the nucleic acid‐based PROTAC design, the common functional group (amido, alkynyl) modified phosphoramidite would be connected to the 5′ site of a single strand nucleic acid sequence via solid phase synthesis. Then, the E3 ligand would be linked to the nucleic acid sequence through condensation or click reaction [78]. In 2020, Ghidini et al. [79] reported the RNA‐based‐PROTACs for degrading RNA‐binding protein (RBP) proteins. Considering the nuclease stability of RNA warhead, the RNA sequence was modified with diastereomeric phosphorothioate and 2′‐O‐methoxyethly. Then, the RNA warhead was connected to the cell permeable LA[Hyp]YI peptide recruiting VHL E3 ligase via condensation reaction (Figure 3B). They validated that the RNA‐based PROTAC molecules effectively degraded the RBP proteins both RBFOX1 and LIN28 through the UPS system in cells at micromolar concentration.

For degrading TFs, Samarasinghe et al. [80, 81] developed the nucleic acid‐based PROTAC called TF targeting chimera (TRAFTAC), which consisted of a dCas9‐specific RNA sequence and a double‐strand DNA sequence. The RNA sequence could bind the dCas9–HaloTag7 fusion protein, which could interact with the VHL‐based Halo–PROTAC molecule for recruiting VHL E3 ligase. The double‐strand DNA sequence was used to recruit TFs. As a proof of concept, they demonstrated that the TRAFTAC effectively degraded tumor‐related nuclear factor kappa B (NF‐κB) and brachyury. Shao et al. [82] also designed the nucleic acid‐based PROTAC for degrading TFs lymphoid enhancer binding factor 1 (LEF1) and ETS‐related gene, respectively (Figure 3C). The selected nucleic acid‐based PROTACs OP‐V1 could effectively degrade LEF1 in vitro (DC_50_ = 25 nM). Importantly, they first validated the effectivity of nucleic acid‐based PROTACs in vivo. Through treating mice with the nucleic acid‐based PROTAC OP‐V1 containing transfection agents (10 mg/kg, tail vein injection), the tumor progression was significantly inhibited by OP‐V1 treatment group compared with control group.

Nucleic acid also could recognize targets through their 2D or 3D structural conformations. For example, G‐quadruplexes, also known as G4, are oligonucleotides that form specific secondary structures. It was stabilized by Hoogsteen hydrogen bonding between the guanine bases in the sequence. These structures played vital roles in regulating gene transcription, repair processes, and replication [83]. Patil et al. [84] developed G4‐based PROTACs for specifically degrading the G4‐binding protein DEAHbox helicase RHAU. The G4‐based PROTACs consisted of a RHAU ligand (a sequence of TT(GGGT)4) and a E3 ligand. After transfection in cells, G4‐based PROTACs effectively induced the RHAU degradation at nanomolar concentration. The other important nucleic acid ligand was aptamer, which could be acquired with systematic evolution of ligands by exponential enrichment (SELEX) technology [85, 86]. The aptamer could leverage their 3D structures to match targets’ structures for binding targets with high specificity and affinity [87, 88, 89, 90]. Zhang et al. [91] designed the aptamer‐based PROTAC ZL216 by connecting DBCO modified‐AS1411 to azide modified‐VHL ligand via the click reaction (Figure 3D). Due to the interactions of AS1411 to nucleolin overexpressed on cancer cells [92], the ZL216 could be internalized selectively into cancer cells. Then, the ZL216 could degrade nucleolin via the UPS system in cancer cells at nanomolar concentration. They also demonstrated that ZL216 (50 µM) could significantly inhibit tumor growth in mice via tail vein injection. Overall, the nucleic acid‐based PROTACs exhibited the unique ability to degrade those POIs without clear binding pockets such as RBP‐binding proteins and TFs.

#### 3.1.3 Peptide as POI Ligand in PROTAC Design

**FIGURE 4:**
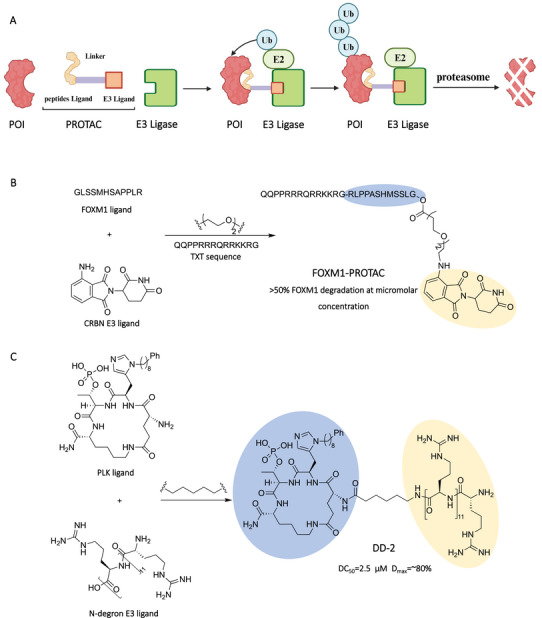
Peptides as POI ligands in PROTACs. (A) The action mode of peptides‐based PROTACs. (B) Design of peptide sequence‐based PROTAC (FOXM1–PROTAC) by connecting the peptide sequence targeting FOXM1 and a CRBN ligand. (C) Design of the cyclic peptide‐based PROTAC (DD‐2) by connecting the cyclic peptide targeting PLK and a N‐dergon E3 ligand. The blue light part and light orange part represent POI ligand and E3 ligand, respectively. The Figure 4A was created in BioRender.com.

Peptides as POI ligands could bind targets through large interaction surfaces [93], which provide the substitutions to bind those proteins with shallow pockets for degradation (Figure 4A) [94]. Particularly, the first PROTAC compound was a peptide‐based PROTAC, in which the phosphopeptide was used to recruit Skpl–Cullin–F box complex, which was first employed for PROTAC‐mediated targeted protein degradation [1]. Generally, by analyzing the key interactions in the crystal structure of endogenous protein and POI, the peptide warhead candidate could be designed to disrupt these interactions. Then, the peptide candidate would be optimized for binding POI with high affinity. Subsequently, the optimized peptide warhead would be linked to the E3 ligand via condensation or click reaction.

The peptide‐based PROTACs usually contain a cell membrane penetrating peptide for facilitating its internalization into cells. For example, Wang et al. [95] designed the peptide‐based PROTAC FOXM1–PROTAC, which was chemically synthesized by linking a FOXM1‐bound peptide antagonist to the E3 Ub ligase‐recruiting ligand pomalidomide and the cell‐membrane‐penetrating peptide (TAT) (Figure 4B). Their results demonstrated that FOXM1–PROTAC could enter cells and induce the degradation of FOXM1, reducing cancer cell viability in a dose‐dependent manner, thereby strongly inhibiting the viability and migration of various cancer cells. In addition, this PROTAC was able to inhibit tumor growth either in MDA‐MB‐231 or in HepG2 cell xenograft mouse models with no detectable toxicity in normal tissues. Ma et al. [96] designed the peptide‐based PROTAC PRTC in which they chose this motif (lysine 266 to valine 286) as the CRPT protein–ligand, which was linked to IYP(OH)Al, via 6‐aminohexanoic acid. A pentapeptide is attached at the C‐terminus of the PRTC to increase cell permeability. Their results showed that PRTC induced the degradation of endogenous cell‐cycle‐related and expression‐elevated protein in tumor (CREPT) proteins, thereby inhibiting tumor migration and progression. Importantly, PRTC showed relatively low toxicity in mice with bearing xenograft tumors. In addition to the peptide sequence as the POI warhead, the cyclic peptide also could be used as the POI warhead in PROTAC design. Gunasekaran et al. [97] designed the peptide‐based PROTAC DD‐2 (Figure 4C). This PROTAC has N‐degron R12 as a UBR‐targeting ligand and a cyclic peptide as a PLK1‐targeting ligand, which were connected by an alkyl linker. Their results showed that DD‐2 degraded polo‐like kinase 1 (PLK1) in HeLa cells in a dose‐dependent manner, with maximal PLK1 degradation achieved at 5 µM, with a *D*_max_ of about 80% and a DC_50_ as low as 2.5 µM. By treating mice with DD‐2, there was a dose‐dependent reduction in tumor size and no significant reduction in body weight.

Overall, various POI ligands (small molecule, nucleic acid, and peptide) were used for PROTACs design, which demonstrated the high protein degradation efficiency and significant therapeutical activity. By analyzing the structural characters of POIs, researchers could select suitable ligands for further PORTAC design.

### 3.2 E3 Ligand in PROTAC Design

**FIGURE 5:**
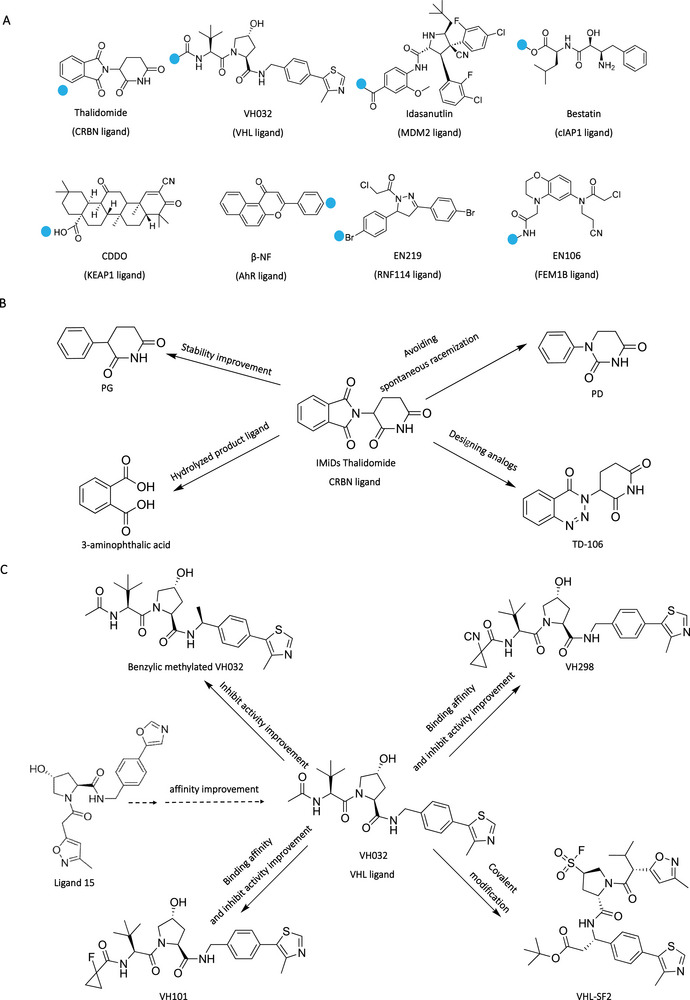
E3 ligands in PROTAC design. (A) Representative E3 ligands in PROTACs, where the blue dot represents the attachment sites. (B) Design and optimization of CRBN E3 ligand Thalidomide. (C) Design and optimization of VHL E3 ligand VH032.

After acquiring the idea POI warhead, it is critical to select the suitable E3 ligand for subsequent PROTAC design. E3 ligase could label targeted proteins with monoubiquitin or various polyubiquitin chains. Only some of these polyubiquitin chains such as K48 could lead to subsequent ubiquitinated protein degradation [98]. Other types of polyubiquitin chains may engage in DNA repair or subcellular localization [99]. Currently, about 270 E3 ligases might be involved in the UPS pathway. However, only limited E3 ligands were discovered for using PROTAC design including CRBN [100], VHL [101], murine double minute 2 (MDM2) [102], cellular inhibitor of apoptosis 1 (cIAP1) [103], Kelch‐like ECH‐associated protein 1 (KEAP1) [104], DDB1‐associated and Cul4‐associated factor 11 (DCAF11) [105], RING finger protein 4 (RNF4) [106], Fem‐1 homologue B (FEM1B) [107], and aryl hydrocarbon receptor [108] E3 ligands (Figure 5A). In the cases of PROTACs against cancers, CRBN ligands and VHL E3 ligands were the two commonly used E3 ligands in PROTACs.

CRBN, the substrate receptor of the CUL4–RBX1–DDB1–CRBN E3 complex, is expressed widely in both physiological and pathophysiological tissues. Immunomodulatory imide drugs (IMiDs) including thalidomide as well as its derivatives (pomalidomide and lenalidomide) have been found to mimic cyclic imide degron for CRBN to induce protein degradation [109]. In the binding mode between IMiDs and CRBN, the glutarimide group occupied the pocket on the CRBN, and the amide on the glutarimide formed a key hydrogen bind with Trp382. The phenyl ring was the solvent‐exposed group on the IMiDs. In the CRBN‐based PROTACs design, the N‐methyl incorporation on the amide of glutarmide of IMiDs could be designed for a negative control PROTAC due to its disruption for the interactions between IMiDs and CRBN, and the phenyl ring was commonly used as a linker attachment site. In 2015, the Bradner group [56] reported the first CRBN‐based PROTAC dBET1. The dBET1 consisted of the BET inhibitor JQ1, linker, and thalidomide, which could completely degrade BRD4 protein at 100 nM within 2 h. In the same year, the Crews group [110] reported the CRBN‐based PROTAC ARV‐825, which was generated by connecting the BRD4 inhibitor OTX015 and pomalidomide with a polyethylene glycol (PEG) linker. The ARV‐825 achieved the highly effective BRD4 degradation (DC_50_ < 1 nM). Subsequently, more and more CRNB‐based PROTACs were developed, which were used for a variety of POIs including BRD2/3, CDK9, CDK4/6, and BTK. It exhibited the broader target spectrum of CRBN‐based PROTACs. Furthermore, the optimization of CRBN ligands also acquired attention (Figure 5B). In the structure of IMiDs, the phthalimide and glutarimide were easily hydrolyzed in vivo, which might damage the degradation efficiency of IMiDs‐based PROTACs. To address this drawback, Rankovic's group [111] designed the novel CRBN ligand named phenyl glutarimide (PG) in which the phthalimide was replaced with the aromatic group. Their results demonstrated that PG had a longer half‐life (*t*_1/2_ > 15 h) than IMiDs, and the PG‐based PROTACs showed a higher degradation efficiency (DC_50_ = 0.87 nM). Subsequently, the C3 of glutarimide in PG was replaced by a nitrogen atom to generate phenyl‐dihydrouracil, which avoids the spontaneous racemization of C3, which could lead to generating the (R)‐enantiomeric ligand with low binding affinity to CRBN [112]. Due to the small size and favorable drug‐like properties, the CRBN ligands become the most used E3 ligands in PROTAC design. However, the neo‐substrates degradation such as GSPT‐1 induced by the molecule glue function of IMiDs might lead to off‐targets toxicity [113].

VHL is a component of a multisubunit E3 ligase complex that contains Elongin B, Elongin C, Cul2, and RBX1 (VBCCR complex), and it naturally recognizes hypoxia‐inducible factor‐1α (HIF‐1α) as its substrate. This recognition leads to the ubiquitination of HIF‐1α and subsequent degradation by the proteasome [101], and the inhibition of HIF‐1α could reverse hypoxia‐induced chemotherapy resistance [114]. Specifically, Ciulli's group [115] demonstrated that the interactions between VHL E3 ligase and HIF‐1α were mediated through hydroxyproline 564 on HIF‐1α. Then, the hydroxyproline was regarded as the starting point for designing VHL E3 ligands. In 2012, Crews's group [116] designed the first small molecule ligand 15 targeting VHL E3 ligase with micromolar binding affinity. Then, they introduced the N‐terminal and C‐terminal modification on ligands respectively to generate ligand 51 with improved binding affinity to VHL. Subsequently, Ciulli's group reported a small molecule VH032 targeting VHL E3 ligase with a higher binding affinity (KD = 185 nM) (Figure 5C). In the binding mode between VH032 and VHL E3 ligase, the presence of the (2S, 4R)‐hydroxyproline core in VH032 was crucial for its binding to VHL, and the inversion of the stereocenter would eliminate the interaction between VH032 and VHL. Based on VH032, Crews et al. [117] developed the first VHL‐based PROTACs for degrading estrogen‐related receptor alpha and RIPK2 at nanomolar concentration in 2015. Then, other optimized VH032 were developed such as VH101 and VH298 [118]. According to the crystal structure of VH032 and VHL E3 ligase, there were solvent exposed points that could be used as linker attachment sites for PROTAC design. For example, the macroPROTAC was designed for stabilizing the active conformation, in which two sites on VHL ligands were linked to POI warhead [46]. Recently, Tate's group [119] designed the covalent VHL ligand VHL‐SF2 in which the sulfonyl fluorides group could covalently bind to the Ser110 on the HIF‐1α. They demonstrated that the two designed PROTACs based on VHL‐SF2 ligand achieved the BRD4 and AR degradation, respectively.

### 3.3 Linker in PROTAC Design

**FIGURE 6:**
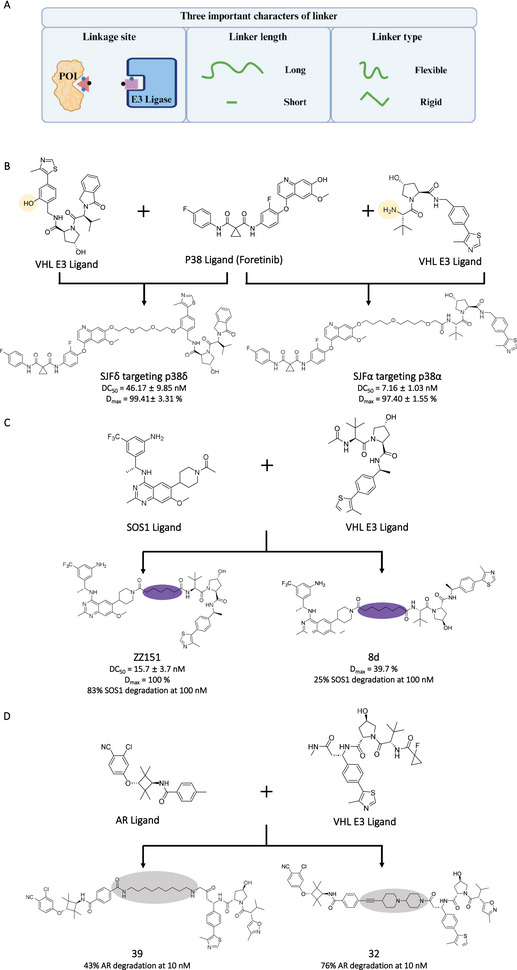
The linkers in PROTAC design. (A) Three important characters of linker in PROTAC design. (B) Different linker attachment sites in E3 ligands in PROTACs (SJFδ and SIFα), where the orange parts represent linker attachment sites. (C) Different linker length in PROTACs (ZZ51 and 8d), where the purple parts represent linkers. (D) Different linker composition in PROTACs (39 and 32), where the gray parts represent linkers. The Figure 6A was created in BioRender.com.

Linker was used to connect the POI ligand and E3 ligand, which played a vital role in the PROTAC‐mediated protein degradation. Generally, the promising linkers could facilitate the formation of ternary complexes induced by PROTACs by adjusting the conformation of POIs and E3 ligases to generate more reasonable interactions [120]. In the past decades, many efforts have been made by researchers for linker design. Specifically, the three characters of linkers were mainly considered (Figure 6A). First, the linker attachment sites between the linkers and POI ligands or E3 ligands would be considered [121]. Through analyzing the crystal structure between protein and ligand, the solvent‐exposed points usually were selected as the linker attachment sites. Attachment sites in PROTACs could influence protein degradation specificity. For example, Crews's group [122] designed two PROTACs (SJFα and SJFδ) with different linker attachments (amine and phenyl attachment sites) on VHL ligands. The SJFα could selectively degrade p38α, while SJFδ prefer to degrade p38δ (Figure 6B). Then, researchers would focus on exploring the length of the linker. On the one hand, a linker that is too short may cause a steric clash between the POI and the E3 ligase, resulting in the disruption of the ternary complex formation. On the other hand, a linker that is too long may cause higher relative motility between the POI and the E3 ligase, thereby reducing the stability of the ternary complex. Typically, the scope of linker length is 3–20 atoms, with short PEG or alkyl linkers were commonly used as a starting point due to their ease of synthesis [123]. For example, Zhou et al. [124] designed the series of SOS1 PROTAC with different linker lengths, which contributed to the dramatic change in degradation efficiency (Compound ZZ151 versus 8d) (Figure 6C). Once the optimal length is determined, the composition of the linker could be optimized to further enhance the degradation efficiency or to improve the pharmacokinetic properties of PROTACs. For example, Han et al. [125] demonstrated that two targeting AR PROTACs with different linker compositions (Compound 39 and 42) exhibited respectively 43% and 76% of AR degradation in LNCap cells at 10 nM (Figure 6D). Furthermore, some relatively rigid linkers such as saturated heterocycles or aromatic rings were also used in linker design [120]. These rigid structures could facilitate constraining the active conformation of PROTACs and introducing additional interactions within the ternary complex to contribute to the degradation activities.

In recent years, the computer‐guided linker design strategy has been used in PROTAC generation [126, 127, 128, 129]. In the computer‐guided approach, the first step involves determining the binding modes between POIs or E3 ligases and their respective ligands, using crystal data or docking simulations. Next, the modeled ternary complex structures of POIs/PROTACs/E3 ligases were generated through protein–protein docking simulations. Subsequently, the modeled complexes underwent restrained minimization to alleviate steric hindrance. Molecular dynamics simulations were used to select more accurate modeled structures for further analysis of interactions and proximity. Based on the ternary complex structures, the linkers could be designed. Finally, the linkers with good performance in computational modeling were validated via wet experiments. Through iteratively modeling and validation, the promising PROTAC compounds could be acquired. For example, Jones’ group [130] designed the highly efficient EML4–ALK PROTAC CPD‐1224 (EML4–ALK^WT^ DC_50_ = 5.4 nM, and EML4–ALK mutation DC_50_ < 100 nM) via computer‐guided optimization strategy. Based on the modeled ternary complex, the flexible methoxy and phenethylamine group were first introduced in linker, and then the linker length and composition were adjusted to acquire CPD‐198. Considering the pharmacology properties, the CPD‐198 was further transformed into CPD‐985. Subsequently, the linker length and rigid were readjusted based modeled ternary complex for acquiring CPD‐1224.

Although many lessons of linker design have been learned, it still has limitations for the linker design. It usually needs to synthesize numerous compounds to screen the promising PROTACs through degradation activity evaluation, which is labor‐ and time‐consuming. Therefore, it is vital to develop the more efficient and precise linker screening strategy for PROTAC design [131].

## 4 AI‐Driven PROTAC Design

Despite the valuable lessons learned from previous PROTACs, designing high efficacy PROTACs within a short timeframe and at a low cost remains a challenge. In the past few years, AI‐based drug discovery acquired witnessed progress [132, 133, 134, 135]. Moreover, AI had good performance in optimizing multiobjectives simultaneously such as binding affinity, selectivity, and pharmacology properties [136]. In this section, we will discuss the application of AI in PROTAC design.

### 4.1 AI‐Driven POI/E3 Ligand in PROTAC Design

In the molecular design of PROTACs, it generally needs to consider three components: POI ligand, linker, and E3 ligand. The small molecule POI/E3 ligand design usually could follow the rule of traditional small molecule drug design. These methodologies included virtual screening [137, 138], high throughput screening [139], and fragment‐based lead discovery [140, 141]. Among nucleic acid‐based ligands, aptamer could be generated through a programmable process based on the SELEX technology [142, 143]. Importantly, AI could accelerate the SELEX process for acquiring the high binding affinity POI ligands in a short time at a low cost [144]. For example, Bashir et al. [145] developed the machine learning‐based aptamer screening strategy. The initial physical experimental data were collected for constructing machine learning model, which then was used for predicting aptamer candidates. This strategy facilitated the high‐affinity aptamer (KD = 1.5 nM) generation. Moreover, AI‐driven POI/E3 peptide ligands were developed for PROTAC design. Ma et al. [31] utilized ProteinMPNN and RFdiffusion for identifying respectively the peptide ligands to bind AR and VHL E3 ligase. Then, these peptide ligands were further used for targeting AR PROTAC design.

### 4.2 AI‐Driven Linker in PROTAC Design

**FIGURE 7:**
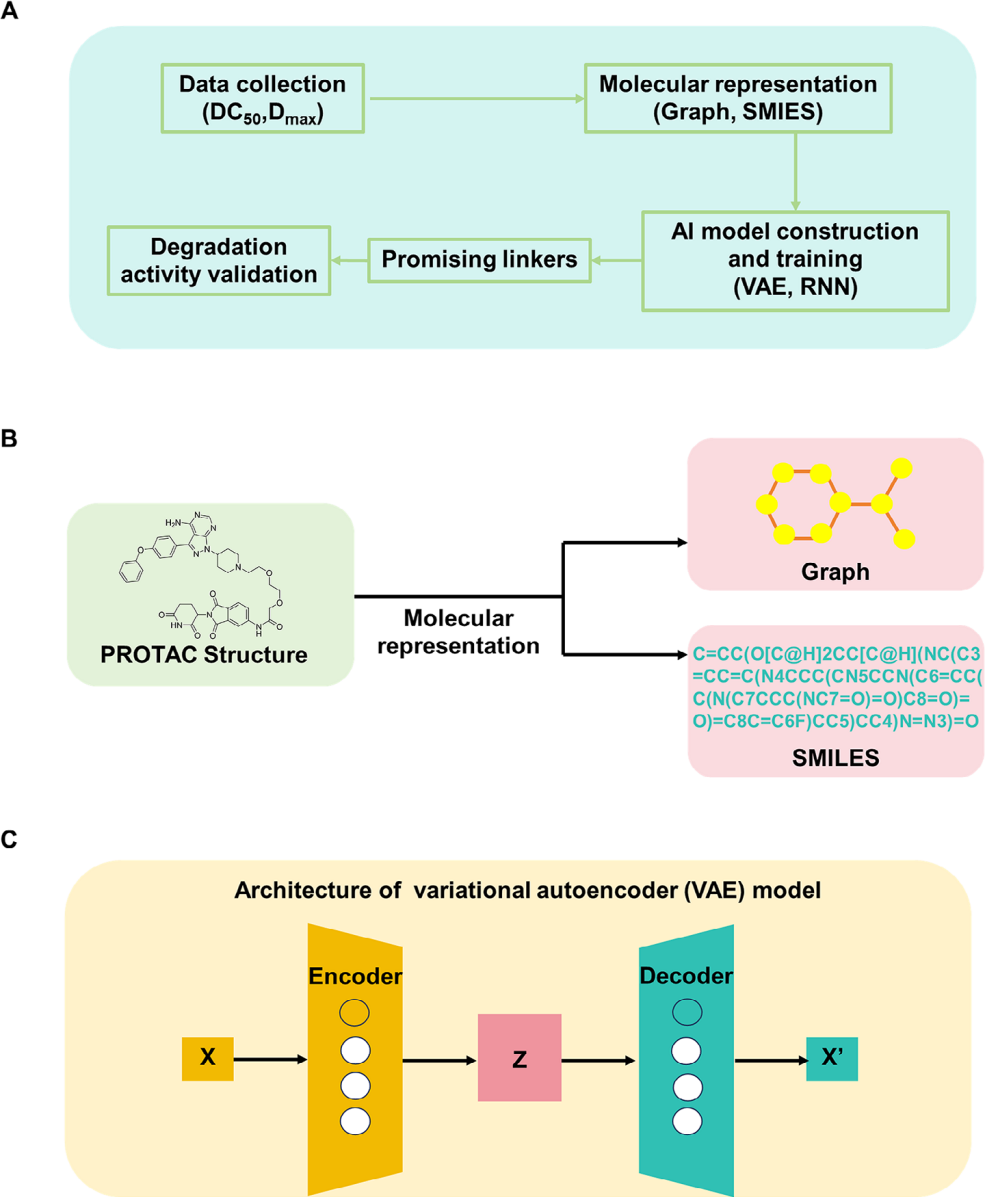
AI‐based linkers in PROTAC design. (A) The flow chart of AI‐based linker design strategies, including the data collection, molecular representation, model construction and training, promising linker output, and degradation efficiency validation. (B) Molecular representations (graph and SMILES) used in AI models. (C) Architectures of VAE model.

It is challenging to screen linkers in PROTACs by using traditional strategy because it might not directly interact with specific targets. Moreover, the linker could not only affect the degradation activities but also generate influences on the pharmacology properties of PROTACs such as cellular uptake [146]. Recently, the AI‐based linker screening strategy was reported, which provided new options for linker design in PROTACs (Figure 7A) [120]. Generally, the process started by collecting a comprehensive dataset of PROTACs, including structures, permeability, stability, degradation efficiency (DC_50_ or *D*_max_). Then, the structures of PROTACs were represented as graphs or simplified molecular input line entry systems (SMILES) (Figure 7B) [147]. Subsequently, the suitable AI modes such as recurrent neural network and variational autoencoder (VAE) (Figure 7C) [148] were constructed and trained. The AI models were subsequently adjusted or optimized to enhance accuracy. Finally, the trained AI models were utilized for linker generation, and the designed PROTACs would be validated in further in vitro and in vivo experiments.

According to the molecular representations, the current AI‐based linker design strategies could be classified into two types. Some AI‐based strategies including DeLinker, 3DLinker, FFLOM, and AIMLinker used graphs as molecular representations, in which atoms were nodes and bonds were edges [149, 150, 151]. For instance, Imrie et al. [151] reported 3DLinker that used an E(3)‐equivariant graph VAE for further task generation, considering the 3D structure and direction of molecules to avoid atomic conflicts. Other AI‐based strategies including PROTAC‐RL, PROTAC‐INVENT, and DRlinker used SMILES as molecular representations [152, 153, 154]. For example, Zheng et al. [154] developed PROTAC‐RL for linker design. They trained the fragment linking model using a large number of compounds with similar chemical space to PROTACs, then these compounds could be fine‐tuned by actual PROTACs. Through this augment process, the >100 times compounds relative to the online PROTAC database were acquired. By pretraining the model with this expanded dataset, their model could learn the chemical space distribution of linkers. Finally, they validated an AI‐driven PROTAC (1) could efficiently degrade BRD4 protein (DC_50_ = 0.116 µM), and it had favorable pharmacokinetics.

Overall, emerging AI‐driven PROTAC design approaches demonstrated promising results. Further efforts should focus on the accuracy and generalizability of modes. Meanwhile, it should integrate more comprehensive properties such as absorption, distribution, metabolism, excretion, and toxicity. Currently, this direction is on the rise, and we believe it could significantly accelerate the development of PROTACs in cancer therapy.

## 5 Recent Applications of PROTACs in Cancer Therapy

To date, PROTACs have been used for various cancer targets degradation with high efficiency. Excitingly, many PROTAC drugs have entered clinical stage and exhibited promising efficacy against cancers. In this section, we will summarize the application of PROTACs in cancer therapy.

### 5.1 PROTACs Targeting Oncogenic Kinases

Oncogenic kinases are a subset of proteins that are part of the kinase enzyme family and play a critical role in promoting cancer development and progression [155]. Targeting oncogenic kinases has become a major focus in cancer research. To date, several PROTACs have been used to degrade oncogenic kinases, including EGFR, ALK, B‐Raf proto‐oncogene serine (BRAF), CDKs, and kinase activity reporters. Zhang et al. [156] reported a targeting EGFR PROTAC (compound 10) that could efficiently degrade EGFR with DC_50_ at 34.8 nM, and significantly induce cell apoptosis in HCC827 cells. In addition, a series of selectively targeting mutant EGFR PROTACs with DC_50_ at nanomolar concentration were reported. Xie et al. [157] designed an alectinib‐based PROTAC (compound 17), which only exhibited antiproliferative activity in ALK‐dependent cell lines rather than ALK fusion‐negative cells. The ALK degradation induced by compound 17 was further validated in vivo, and the reduction in tumor weight induced by compound 17 reached 75.82%. Alabi et al. [158] reported the targeting mutant BRAF PROTAC (SJF‐0628), which exhibited low nanomolar degradation efficiency for all classes of BRAF mutants, but spare degradation for WT BRAF. The SJF‐0628 also exhibited higher antiproliferative activity than the inhibitor vemurafenib. PROTACs targeting CDKs including CDK4/6/12 had been reported. For example, Jiang et al. [159] designed a targeting CDK12 PROTAC BSJ‐4‐116, which could selectively degrade CDK12, leading to premature cleavage and polyadenylation of DDR genes.

### 5.2 PROTACs Targeting Epigenetic Regulators

Dysregulation of epigenetic regulators is commonly linked to cancer, and these regulators are essential for regulating cellular processes and gene expression patterns [160]. So far, several epigenetic regulators were degraded by PROTACs, such as BRD4/9, histone deacetylase (HDAC), and the p300/CBP‐associated factor (PCAF) and the general control nonderepressible 5 (GCN5) [161]. There were many typical PROTAC compounds (ARV‐825, dBET1, MZ1, QCA570, and so on) that were designed for BRD4, which exhibited high effective degradation ability for BRD4 with DC_50_ at nanomolar concentration, leading to inhibition of downstream oncogene expression. Smalley et al. [162] identified the PROTAC compound 9 for HDAC1/2 degradation with submicromolar DC_50_ value, and compound 9 could enhance apoptosis in HCT116 cells. Bassi et al. [163] reported the first targeting PCAF/GCN5 PROTAC (GSK699), which could efficiently degrade PCAF/GCN5 and modulate the expression of multiple inflammatory mediators.

### 5.3 PROTACs Targeting Antiapoptotic Proteins

Cancer cells could enhance some specific antiapoptotic protein expression to escape programmed cell death [164]. Therefore, antiapoptotic proteins have been regarded as attractive druggable targets in cancer therapy. The classical antiapoptotic proteins included BCL2 family members and inhibitor of apoptosis proteins (IAPs) family members, which had been successfully degraded by various PROTACs [165]. Zhou's group [166] reported an effective BCL‐XL PROTAC (DT2216) with DC_50_ at 63 nM, which achieved significant inhibition of tumor growth and it could not lead to severe platelet reduction. Subsequently, Zhou's group [167] further designed the more effective BCL‐XL PROTAC (XZ739), which could strongly induce BCL‐XL degradation (DC_50_ = 2.5 nM) and significantly inhibit various cancer cell proliferation. Moreover, based on the computational modeling of ternary complex induced by PROTAC, Zhou's group [168] generated a targeting BCL‐XL and BCL‐2 dual PROTAC (753b), which exhibited improved antitumor activity. Papatzimas et al. [169] reported a targeting antiapoptotic protein myeloid cell leukemia 1 (MCL1) PROTAC (dMCL1‐2), which could efficiently degrade MCL1 at nanomolar concentration and activate the cellular apoptosis machinery. Park et al. [170] first reported a targeting cIAP1/2 and XIAP PROTAC (TD‐1092), which could rapidly reduce cIAP1/2 and XIAP levels at submicromolar concentration, leading to cell apoptosis. Ng et al. [171] reported a targeting IAPs PROTAC (compound 9), which enabled effective IAPs degradation (DC_50_ = 2.4 nM for cIAP1, DC_50_ = 6.2 nM for cIAP2, and DC_50_ = 0.7 nM for XIAP) and exhibited significant inhibition for the viability of cancer cells.

### 5.4 Clinical Advances of PROTACs in Cancer Therapy

In the past few years, PROTAC technology acquired significant advances in cancer therapy. Particularly, many PROTACs entered a late stage of preclinical trials, and some of them entered phase 1/2/3 clinical trials [25]. Arvinas developed ARV‐110, the first‐in‐class AR‐PROTAC molecule [172]. In preclinical studies, ARV‐110 effectively degraded the AR protein in prostate cancer cells, with a DC_50_ concentration in the nanomolar range in vitro. In clinical studies, ARV‐110 treatment group showed tumor reduction in patients, with 46% of them experiencing a decline of over 50% in prostate‐specific antigen levels. Notably, the phase 1/2 dose escalation clinical trial reported only one adverse event among 18 patients, indicating a favorable safety profile for ARV‐110 in the treatment of castration‐resistant prostate cancer. A targeting ER PROTAC named ARV‐471 was also developed by Arvinas [173]. In preclinical studies, ARV‐471 effectively degraded ER in metastatic breast cancer cells at nanomolar concentrations in vitro and reduced ER expression in metastatic breast cancer models in vivo. In clinical studies, it has been reported that patients with an administration of 360 mg dose of ARV‐471 did not experience grade 3 or 4 treatment‐related adverse events. Nurix Therapeutics has developed NX‐2127, a CRBN ligand‐based PROTAC that could simultaneously degrade BTK and IKZF1/3 proteins [174]. In preclinical studies, NX‐2127 efficiently degraded BTK protein in B‐cell lymphoma cells, with a DC_50_ value of <5 nM, and degraded IKZF1/3 protein in human T cells, with a DC_50_ value of 25 nM. Importantly, NX‐2127 significantly inhibited the proliferation of B‐cell lymphoma cells with ibrutinib resistance. The NX‐2127 exhibited superior tumor‐inhibiting abilities compared with ibrutinib. Moreover, Kymera designed a targeting MDM2 PROTAC KT‐253, which exhibited significantly higher antitumor activity than clinical MDM2 small molecule inhibitors. CFT1946 was a targeting BRAF^V600X^ PROTAC developed by C4 Therapeutics. They demonstrated that CFT1946 could selectively degrade BRAF^V600X^, inhibit the MAPK signaling pathway, and inhibit tumor progress. Other PROTACs ongoing clinical trials were listed in Table 1 [26].

**TABLE 1:** Ongoing clinical trials of PROTACs in cancer therapy.^a^, ^b^

Clinical trial NCT no.	Phase	PROTAC	Rout of administration	POI	E3 ligase	Indications	Sponsor
NCTO3888612	II	ARV‐110	Oral	AR	CRBN	PC	Arvinas
NCTO4428788	I	CC‐94676	Oral	AR	CRBN	PC	Bristol Myers Squibb
NCTO5241613	I	AC0176	Oral	AR	Undisclosed	PC	Accutar Biotech
NCTO5252364	I	HP518	Oral	AR	Undisclosed	PC	Hinova
NCTO5067140	I	ARV‐766	Oral	AR	Undisclosed	PC	Arvinas
NCTO4072952	II	ARV‐471	Oral	ER	CRBN	BC	Arvinas/Pfizer
NCTO5080842	I	AC0682	Oral	ER	CRBN	BC	Accutar Biotech
NCTO4830137	I	NX‐2127	Oral	BTK	CRBN	B cell malignancies	Nurix Therapeutics
NCTO5131022	I	NX‐5948	Oral	BTK	CRBN	B cell malignancies and autoimmune diseases	Nurix Therapeutics
NCTO5006716	I	BGB‐16673	Oral	BTK	Undisclosed	B cell malignancies	BeiGene
NCTO4861779	I	HSK29116	Oral	BTK	Undisclosed	B cell malignancies	HAISCO
NCTO5225584	I	KT‐333	i.v.	STAT3	Undisclosed	Liquid and solid tumors	Kymera
CTR20222742	IND‐e	KT‐253	Undisclosed	MDM2	Undisclosed	Liquid and solid tumors	Kymera
	IND‐e	CFT1946	Oral	BRAF^V600x^	Undisclosed	Melanoma, CRC, NSCLC	C4 Therapeutics
	IND‐e	CFT8919	Oral	EGFR^L858R^	CRBN	NSCLC	C4 Therapeutics

Abbreviations: BC, breast cancer; CRC, colorectal cancer; NSCLC, non‐small cell lung cancer; PC, prostate cancer.

^a^ Information from the *Clinicalrials.gov* database. with the keywords: “PROTAC,” “cancer,” and “degrader.”

^b^ Information from the *Chinadrugtrials.org.cn*.

## 6 Challenges of PROTAC Design and Development in Cancer Therapy

Despite of rapid development of PROTAC technologies, it still remains challenges for further translation [175]. In this section, we will discuss the main challenges of PROTACs in cancer therapy including poor cell permeability, off‐tissue toxicity, off‐target effects, and acquired resistance. Particularly, we will present some novel PROTACs for addressing these issues, and hope to provide some valuable insights for future development of PROTACs.

### 6.1 | Poor Cell Permeability

PROTAC activity against intracellular targets depended on permeability, which generally decreased sharply as molecular weight increases. Some PROTACs have a high molecular weight exceeding the “rule‐of‐5” threshold of 500 Da, which is a guideline for orally active small‐molecule drugs. Interestingly, these PROTACs exhibited intracellular targets degradation activity. It might be resulted from the unique catalytic mode of action, which allowed substoichiometric PROTACs to degrade intracellular POIs. Moreover, the other reasons might be resulted from the linker‐mediated shielding function for polar surface area in PROTAC [176]. Currently, there are some optimization strategies focusing on the permeability of PROTACs. The replacement of a nonpolar bond with a polar bond reduced the hydrogen bonds and the polar surface area, which could improve the membrane‐permeability of PROTACs. In addition, Lebraud et al. [177] designed in‐cell self‐assembly PROTACs to address the poor cell permeability issues. Two low molecule weight ligands (a tetrazine linked thalidomide derivative and TCO tagged JQ1 ligand) could respectively enter cells and then form active targeting BRD4 PROTAC in cells via click reaction for BRD4 degradation. Other factors like lipophilicity/solubility also affected the cell permeability. Furthermore, various PROTAC delivery agents (such as nanoparticle, aptamer, and cell‐penetrating peptide) were developed to improve drug intracellular accumulation [178]. For example, Chen et al. [179] reported a prefused ARV771 loading on lipid‐like nanoparticles could improve the cellular uptake of prefused ARV771, leading to efficient POI degradation.

### 6.2 Off‐Tissue Toxicity

Despite the rapid development of PROTAC technology, most of the PROTACs could degrade POIs both in cancer and normal cells, leading to off‐tissue toxicity when POIs played vital roles in normal cells. For example, ARV‐110, which was designed for treating prostate cancer, has demonstrated effectiveness but also highlighted the need for preventing damage to healthy tissues and minimizing adverse effects [180]. Therefore, cancer‐selective POI degradation was important in PROTAC design for their clinical translation. One strategy leveraged the tumor microenvironment (TME) in cancers, which serves as triggers for endo‐stimuli responsive PROTACs to achieve precise protein degradation [181]. The TME contains various unique conditions such as low pH, high levels of redox potential, and overexpressed intracellular enzymes [182]. By incorporating TME‐responsive elements into PROTACs, these unique conditions could specifically trigger precise protein degradation in cancers [183, 184, 185, 186]. For example, Cheng et al. [187] developed a hypoxia‐activated PROTAC (compound 1) by incorporating a hypoxia‐activated leaving group into the POI ligand for the degradation of EGFR. Western blot results demonstrated that compound 1 induced substantially higher EGFR degradation in hypoxia compared with nonhypoxia in vitro. Another strategy leveraged cancer‐selective drug delivery system [188, 189, 190]. For instance, Liu et al. [188] designed folate‐caged PROTAC folate–ARV‐771, which preferentially degraded BRDs in cancer cells in a folate receptor‐dependent manner.

### 6.3 Off‐Target Effects

Generally, PROTACs were more selective when compared with small molecule inhibitors due to the strictness and ingenuity of inducing a stable ternary complex. Bondeson et al. [29] reported that a nonselective kinase inhibitor‐based PROTAC could selectively degrade <15 kinases even though it could bind to over 50 kinases. They demonstrated that this selectivity has relied on protein–protein interactions for forming stable ternary complexes. However, the formation of the ternary complex would be inhibited when PROTACs were treated at high concentrations, and the PROTACs/POIs or PROTACs/E3 ligases binary complex would be formed. This phenomenon in most of the PROTACs was known as the “Hook effect” [191]. The PROTAC/E3 ligase binary complex resulting from the Hook effect could recruit some low‐affinity targets, leading to off‐targets degradation [192]. In addition, some CRBN‐based PROTACs have been proven they lead to the degradation of natural substrates of CRBN including IKZF1/3, ZNF827, and ZFP91, which may lead to undesirable effects due to the function of these substrates for normal homeostasis [10, 193]. Mass spectrometry‐based proteomics could be a great tools to evaluate the degradation selectivity and safety of PROTACs [194]. Utilizing exo‐stimuli conditions such as light to control POI degradation could alleviate off‐target effects [195, 196, 197, 198, 199, 200]. By incorporating exo‐stimuli elements into PROTACs, the specific exo‐stimuli can induce precise protein degradation in cancer cells. For example, Deiters et al. [197] designed photo‐caging PROTACs for controlling ERRα degradation. They connected a bulky photolabile group, (7‐diethylaminocoumarin‐4‐yl) methoxycarbonyl, to the hydroxyl group of the proline moiety in the ERRα targeting PROTAC through a carbonate linkage. In the absence of UV radiation (*λ* = 405 nm), MCF‐7 cells treated with ERRα PROTAC 2 showed no significant increase in luciferase signal. However, in the presence of UV radiation, luciferase expression was restored to levels comparable to those achieved with the original PROTAC treatment.

### 6.4 Acquired Resistance

Innate and acquired drug resistance usually occurs in cancer therapy, which could be mediated by mutations, changes in gene expression, and posttranslational protein modifications [201]. With the applications of PROTACs in cancer therapy, it was necessary to consider the drug‐acquired resistance. Zhang et al. [202] demonstrated that resistance would occur in cancer cells after the chronic treatment of VHL‐ or CRBN‐based PROTACs. In their studies, the OVCAR8 cell lines were exposed to VHL‐ or CRBN‐based BET‐PROTACs over 4 months. Then, two resistant derivatives O1R and O3R cell lines were generated. The O1R exhibited a 87.4‐fold higher IC_50_ for VHL‐based PROTAC (ARV‐771) than parental OVCAR8 cell lines. The O3R exhibited a 41.0‐fold higher IC_50_ for CRBN‐based PROTAC (ARV‐825) than parental OVCAR8 cell lines. They observed that removing the PROTACs for 2 months could not diminish resistance, indicating that this resistance might be resulted from the stable genetic changes. Importantly, their genetic analysis revealed that the resistance mainly resulted from the genomic alterations that compromised the core components of the respective E3 ligase complex. Shirasaki et al. [203] used functional genomics to investigate the mechanism of resistance for PROTACs in cancer cells. Their results showed that the resistance to BET or CDK9 targeting PROTACs in myeloma cells was mainly mediated by preventing rather than adapting the POIs degradation, which also involved the function loss of the E3 ligase or regulators of the respective CRL complex.

## 7 Conclusion and Future Directions of PROTAC in Design and Development in Cancer Therapy

In past decades, PROTAC technology acquired rapid development and exhibited great potential for cancer therapy. This novel therapeutic demonstrated that targeted POIs degradation could address the bottleneck for traditional inhibitor‐based drugs [204]. It did not need to occupy the active sites on POIs for further activity inhibition. Moreover, the catalytical degradation mode could allow it exhibited significant efficacy at low concentration. Particularly, PROTAC paved the new way for targeting those “undruggable” POIs. Currently, a large amount of small molecule‐based PROTACs were designed for cancer‐related POIs. Among these POIs, the majority of targets were druggable. It was intractable for small molecule‐based PROTACs to degrade those POIs without specific binding pockets. Therefore, expanding the accessibility for POI ligands for those intractable POIs was important for PROTAC technology. Recently years, some emerging PROTAC modalities such as nucleic acid‐based PROTACs and peptide‐based PROTACs were developed. These modalities could selectively recruit those intractable POIs through complementary structural characters or large binding faces for further degradation via UPS system. On the one hand, the POI ligands (nucleic acid or peptide sequence) could be generated via mimicking natural POI binding partners. The critical binding motifs on natural POI binding partners could be refined and optimized as POI ligands. On the other hand, the POI ligands (nucleic or peptide aptamers) could be generated via established screening technology such as SELEX. Although nucleic acid‐based PROTACs and peptide‐based PROTACs exhibited unique capacity for targeting undruggable targets, their druggability might be further considered. Compared with small molecule, nucleic acid or peptide was more easily degraded in vivo. In addition, the poor permeability issues of nucleic acid or peptide were challenging in their clinical translation. Generally, the chemical modification or delivery system was necessary in nucleic acid or peptide‐based PROTAC design.

To design an effective PROTAC, the screening of linker and E3 ligand was critical. Based on the expression level of E3 ligases, researchers could select corresponding E3 ligands for further PROTAC design. Currently, VHL and CRBN ligands were commonly used in small molecule‐, nucleic acid‐, and peptide‐based PROTACs. The PEG and alky linkers were widely used in preliminary PROTAC design. According to activity validation and structural insights from crystal structures or computational modeling, the linker would be further optimized for improving the degradation efficiency. However, the rational linker design still was a bottleneck in PROTAC design. One of reasons was that linker might be not interacted with specific binding motif, which could contribute to degradation via adjusting protein–protein interfaces and conformations [205]. Therefore, it was difficult to acquire the specific structure‐activity relationships. Overall, the obtainment of promising linker was a trial‐and‐error process in PROTAC design.

AI‐based drug discovery acquired extensive attention recent years, which could accelerate the drug discovery in vast chemical space. Particularly, the emerging AI technology such as Alphafold3 could accurately predict the protein structures [206]. It allowed us to select suitable ligands according to structural characters of proteins, which might be the small molecules, nucleic acids, or peptides. Because PROTAC did not need to occupy the active site on the proteins, so the binding affinity and specificity of POI ligands should be critical in this process. Although some substantial progress of AI‐based PROTAC design was reported including prediction of degradation efficiency, structural design, and pharmacokinetics optimization, the future efforts should be focused on the accuracy of model and addressing challenging druggability issues. Moreover, one of challenges for AI‐based PROTAC model was data scarcity, which could lead to the overfitting and limited generalization ability of model [30]. To further accelerate the PROTAC development, the following three directions could be considered:

### 7.1 Expanding the E3 Ligase Toolbox

In the past two decades, the various E3 ligases were successfully engaged in PROTAC‐mediated targeted protein degradation. However, the E3 ligase toolbox still is insufficient and limited. Currently, only CRBN and VHL E3 ligases are widely used in PROTACs‐mediated protein degradation. It could not avoid some potential problems such as acquired resistance and off‐target issues. How to expand the new E3 ligases was important in future PROTAC development. So far, some studies have shown potential E3 ligases that could be used for protein degradation by analyzing online datasets. Liu et al. [207] reported a comprehensive bioinformatic analysis for potential E3 ligases. On their website, researches could search matched E3 ligase for PROTAC design. Based on the proteomics data from the Human Protein Atlas, Schapira et al. [205] demonstrated that 24 E3 ligases were detected in at least 90% of cell and tissue types tested. Furthermore, they analyzed that 20 E3 ligases had a narrow expression window, which may be used as the tissue‐selective E3 ligases for further PROTACs design. To determine the abundances of E3 ligase protein levels, mass spectrometry‐based proteomics technologies including both data‐dependent and data‐independent acquisitions could be used [208]. Moreover, comprehensive structure analysis and in vitro ubiquitinated activity of identified E3 ligases need to be investigated. The collaborative efforts between chemical biology and AI tools such as Alphafold 3 were necessary in this filed.

### 7.2 Novel PROTAC Modalities

Despite of rapid development of PROTACs, effective PROTACs still involve complicated design processes such as ligand optimization and linker screening. Therefore, exploring novel PROTAC modalities was necessary. Introducing new modalities such as nano materials could simplify the PROTAC's design. For instance, Song et al. [209] reported a split‐and‐mix liposome PROTAC platform. Both the POI ligand and E3 ligand were conjugated to DSPE‐PEG2000, and then these conjugates were directly used for preparing liposome‐based PROTAC. Their results showed that this platform could efficiently degrade MEK1/2 and ALK proteins in cancer cells, and exhibited obvious tumor inhibition in vivo. In addition to liposomes, other materials such as DNA frameworks, hydrogels, and polymeric nanoparticles might be used in novel PROTAC modalities design in the future. These materials had self‐assemble characters, multiple attachment sites, and biocompatibility [210, 211, 212, 213]. In the future, by combining these novel materials, it is promising to design the universal PROTAC‐based targeted protein degradation platform.

### 7.3 Alternative Degradation Pathways

While PROTAC technologies pioneered the targeted protein degradation by harnessing the UPS pathway, the reliance on UPS may limit their application to those proteins that were amenable to ubiquitination and proteasomal degradation. Therefore, exploring the alterative degradation pathways was very important, which could expand the landscape of POIs and provide potential therapeutics for cancer therapy. In the past years, various targeted protein degradation strategies have been developed [214]. Lysosome‐targeting chimera (LYTAC) was a promising strategy to degrade membrane or extracellular POIs. LYTAC usually consisted of a POI ligand, a linker, and a lysosome‐targeting ligand. It could recruit POI and lysosome‐targeting ligand and then transport POI into the lysosome for degradation. Currently, various types of LYTACs including antibody‐, peptide‐, and aptamer‐based LYTACs [215, 216, 217] were developed, and many POIs inaccessible for PROTACs such as secreted cytokines, growth factors, and cell surface receptors were successfully degraded by LYTACs [218]. Autophagy‐targeting chimera was a bifunctional molecule consisting of a POI ligand linked to an autophagy‐targeting tag such as a guanine derivative [219]. Once POI was tagged, it would be engulfed by autophagosome and then delivered to the lysosome for degradation. These emerging targeted protein technologies greatly expanded the target landscape of PROTACs and provided potential therapeutics in cancer therapy. Although these technologies were in their infancy, they had great potential with leveraging advancing research technologies such as AI and multiomics.

## Author Contributions

H.L. and Y.T. wrote the manuscript, and R.A. prepared the figures and tables. B.Z., Y.M., and G.Z. devised the project and supervised the preparation of the manuscript. All authors have read and approved the final manuscript.

## Ethics Statement

The authors have nothing to report.

## Conflicts of Interest

All authors declare no conflicts of interest.

## Data Availability

The authors have nothing to report.
